# Cannabis Use among Older Persons with Arthritis, Cancer and Multiple Sclerosis: Are We Comparing Apples and Oranges?

**DOI:** 10.3390/brainsci11050532

**Published:** 2021-04-23

**Authors:** Brian Kaskie, Hyojung Kang, Divya Bhagianadh, Julie Bobitt

**Affiliations:** 1 College of Public Health, University of Iowa, Iowa City, IA 52242, USA; brian-kaskie@uiowa.edu (B.K.); divya-bhagianadh@uiowa.edu (D.B.); 2 College of Applied Health Sciences, University of Illinois at Urbana Champaign, Champaign, IL 61820, USA; hyokang@illinois.edu; 3 College of Medicine, University of Illinois at Chicago, Chicago, IL 60612, USA

**Keywords:** multiple sclerosis, older adults, opioids, cannabis

## Abstract

Although researchers have identified medications that relieve symptoms of multiple sclerosis (MS), none are entirely effective and some persons with multiple sclerosis (PwMS) use alternatives. Our study compared cannabis use among PwMS (N = 135) and persons diagnosed with arthritis (N = 582) or cancer (N = 622) who were age 60 and older, enrolled in the State of Illinois Medical Cannabis Program, and invited to complete a survey fielded between June and September, 2019. We used logistic regression to identify significant differences in self-reported effects of cannabis on psychological wellbeing, quality of life, and three behavioral outcomes, and we also considered effects of past year opioid use relative to these outcomes. We found that the majority of individuals from all groups used cannabis to address pain and improve quality of sleep. While PwMS reported lower baseline levels across all five outcomes, we found that the reported effects of cannabis were largely comparable across the groups. We also found that cannabis benefitted persons with sleep and digestive issues regardless of condition, whereas persons who used opioids in addition to cannabis were less likely to experience an improvement in any of the outcomes. This comparative evaluation suggests that cannabis’ effects are not specific to MS, arthritis, or cancer as much as they impact processes common among these distinct conditions. We also found evidence that cannabis may be a viable alternative to opioids for those with these conditions and experiencing pain.

## 1. Introduction

Nearly one million Americans currently live with multiple sclerosis (MS) [1]. With no known cure or treatment alternative that effectively stops disease progression, MS stands apart as an insidious, chronic, and disabling condition. Persons with multiple sclerosis (PwMS) experience visual, somatosensory, and motor pathway impairments as well as affective, cognitive, and psychiatric disturbances [2,3]. MS is not considered fatal. Many PwMS may reach anticipated pre-morbid life expectancies progressing through different symptomatic profiles, alternating between disease progression and remission [2]. While researchers have identified a number of prescription medications that may help slow the progression of disability, relieve symptoms, and deter relapses, none of these are entirely effective [4]. Some PwMS use complementary and alternative therapeutic approaches to manage their symptoms [5] and, in this paper, we examine the growing number of PwMS who are using cannabis to reduce pain and other undesirable symptoms.

### 1.1. Background

In 2017, a national survey of PwMS revealed that forty-seven percent of respondents had considered using cannabis to treat their symptoms, twenty-six percent actually had used cannabis for symptom management, and sixteen percent currently were using cannabis [6]. Other surveys [7,8,9,10] suggest that current use rates may differ across ethnic groups and be higher within those states that have legalized cannabis for medical purposes and identified MS as a qualifying condition for program participation. While these prevalence rates remain somewhat indefinite, PwMS are up to ten times more likely to use cannabis than persons without MS and researchers have established that cannabis use among PwMS is driven by age, gender, prior history of cannabis use, and disability [8,9].

One of the primary reasons that PwMS use cannabis is to control spasticity, and the National Academy of Medicine [11] identified a sufficient amount of evidence to endorse such medical use as clinically efficacious. Other researchers suggest that PwMS use cannabis to reduce symptoms of pain [7,8]. Chronic pain is experienced by at least half of PwMS, and cannabis use has been associated with self-reported pain improvement across several studies. Yadav, Bever, and Bowen [5] reported that two of every three randomized and controlled clinical studies involving PwMS indicated that cannabis (or cannabis products) provided at least modest amounts of pain relief. Besides decreased spasticity and lower levels of self-reported pain, researchers have examined how cannabis may impact sleep, digestive functions, and balance among PwMS [7,8].

In our previous work, we have focused on the emerging public health concern with the increasing use of cannabis among all persons over sixty years old. In the state of Illinois, for example, older persons constituted the largest group of participants enrolled in the state medical cannabis program and we found that those participants diagnosed with multiple sclerosis were among the largest groups of individual program participants. In addition, we observed large numbers of older participants who were diagnosed with arthritis and cancer [12]. Previous work also suggests that older persons with arthritis or cancer who use cannabis significantly differ from those who do not—just as PwMS who use cannabis differ significantly from those who do not [13]. At this point, a comparative evaluation among different groups of older cannabis users may offer more incisive insights about general and disease-specific effects.

On one hand, researchers have determined how cannabis acts upon the endocannabinoid system and can affect immune system function. For example, Kleckner et al. [14] contend that cannabis operates as an anti-inflammatory and, in so doing, reduces pain regardless of underlying medical condition. Researchers also have identified how cannabis affects psychoactive function and, in so doing, may elevate mood or sense of wellbeing regardless of underlying medical condition. This leads us to suspect that older PwMS may experience comparable outcomes with cannabis users diagnosed with arthritis or cancer.

On the other hand, Almoge-Hazan and Or [15] suggest that cannabis may impact disease-specific processes and identified current clinical trials examining how cannabis use impacts movement among persons with auto-immune diseases such as multiple sclerosis or rheumatoid arthritis. They also discussed how cannabis may alter pathogenic processes that advance tumor progression and digestive dysfunction among persons with cancer and identified current clinical trials examining how cannabis-based therapies may affect cancer-specific symptoms including cachexia, nausea, and tumor progression.

A comparative evaluation among different groups of older cannabis users also may help to differentiate self-reported outcomes experienced by older adults who use cannabis for different reasons. In our previous work, we found that older adults who use cannabis experience several desirable outcomes, such as reduced pain, improved sleep, and increased quality of life, and found some evidence that these outcomes were independent of each other—perhaps because they were shaped differently relative to underlying diagnosis or baseline status [16].

Moreover, the co-occurring use of medical cannabis and prescription opioids has emerged as a public health concern [17]. One recent study of National Survey of Drug Use and Health data showed that over 50% of older cannabis users also used prescription pain relievers [18]. However, it remains unclear if PwMS who experience pain combine cannabis and prescription opioids to a greater or lesser extent than others who also experience pain and use cannabis and prescription opioids together [19]. Are PwMS with pain who use cannabis more or less likely to obtain opioids than those older adults with arthritis or cancer with similar levels of pain? Do they experience better outcomes?

### 1.2. Research Objectives

To gain a better understanding of how cannabis may selectively impact PwMS relative to cannabis users with other chronic, disabling conditions, we collected survey data from adults age 60 and older who participated in the Illinois Medical Cannabis Program (IMCP). With these data, we identified significant differences across groups of individual IMCP participants, including PwMS and those diagnosed with arthritis or cancer (two of the most commonly identified conditions among medical cannabis program participants). We then applied an integrated model developed in our previous research [20] and regressed a range of self-reported outcomes (psychological wellbeing, quality of life, productivity, exercise, and social participation) onto individual characteristics (e.g., age, gender, education), health conditions (arthritis, cancer, or MS; digestive problems) and symptoms (e.g., pain, sleep, spasticity), and use of prescription opioids in the past year. We considered the first two outcomes a reflection of an individual’s psychological status that could be impacted by cannabis regardless of diagnostic group (through CB1 receptors) [14,15]. The latter three consist of behaviors that may be impacted differently as persons with arthritis and MS may respond differently to the anti-inflammatory effects (via CB2 receptors) of cannabis that may make physical movement less painful [15]. Such modeling may help us to: (a) better distinguish among PwMS participating in a legal medical cannabis program, (b) compare a range of self-reported outcomes between PwMS and those diagnosed with arthritis and cancer, and (c) determine if prescription opioid use occurs more or less frequently among PwMS and if co-occurring use is related to any of these outcomes.

## 2. Materials and Methods

For this study, we relied on state-wide, cross-sectional, electronic survey data from individuals aged 60 and above who were actively enrolled in the IMCP at the time of survey distribution between June and September 2019. Because of the lack of attention to the increasing use of cannabis among older persons, we only surveyed individuals aged 60 and older who, at the time of the survey, relied on the IMCP as the only legal way to access cannabis. While our survey included individuals with any qualifying condition, this analysis focused on those with arthritis, cancer, and MS—which were among the largest groups of older adults participating in the IMCP [21]. Data were collected and managed using REDCap, a secure, web-based application designed to support electronic data capture [22]. The project was approved by the Institutional Review Board at the University of Illinois at Urbana-Champaign, and all participants reviewed a consent document before completing the survey.

### 2.1. Survey Data

From June to October of 2019, the Illinois Department of Public Health sent three electronic invitations to 16,584 persons aged 60 and over who were enrolled in the IMCP. The survey consisted of 88 questions asking about individual characteristics, health status, past-year use of cannabis and opioids, and outcomes. These questions were selected from our previous survey work and many of these have been associated with cannabis use among older persons. We started by asking participants to rate their education and financial status using a categorical scale and then combined this information into binary groups (i.e., college or higher, financially secure vs. not financially secure). Since the IMCP offers cannabis to individuals with any 1 of 45 different diagnosed conditions, we asked, “For what medical conditions do you use cannabis?” and persons included in this study selected arthritis, cancer, or multiple sclerosis. We measured self-reported level of pain in the last 30 days on a 0–10 scale, with zero being no pain and 10 being the worst pain ever. We also asked about reasons for cannabis use, including sleep issues, digestive problems, and spasticity. We asked participants if they used opioids in the past year. We measured how cannabis use impacted psychological wellbeing, overall quality of life, productivity (To what extent are you able to grocery shop?), participation in exercise, and social satisfaction using a 5 pt. Likert scale, where 1 is poor and 5 is excellent. We also asked participants if cannabis made it worse, no change, or made it better. Because of the low rates of negative outcomes reported, we grouped “make it worse” and “no change” as non-positive and coded as 1, and we coded “make it better” as 0.

### 2.2. Sample

We obtained 4066 survey responses from persons over age 60 who were currently enrolled in the IMCP and used cannabis in the past year. Among these, 135 identified themselves as being qualified for the IMCP based on a certified diagnosis of MS. We also identified participants who were qualified based on diagnoses of arthritis (N = 586) and cancer (N = 622). Individuals who were not over the age of 60, did not report their age, had other conditions, or only used cannabis for recreational purposes were excluded from this study. Persons who reported having both MS and arthritis, MS and cancer, or arthritis and cancer were not included in the analysis.

### 2.3. Analysis

We performed univariate analysis using chi-square and t-tests to identify significant differences among groups. We then examined the relationship between cannabis use and self-reported psychological wellbeing, quality of life, productivity, exercise, and social satisfaction using logistic regression to determine how main effects (group status) are independently associated with outcomes while controlling for covariates. We included the measure of past-year opioid use in the logistic regression models to estimate effects of co-occurring use, and we tested for an interaction effect between diagnostic status and past-year opioid use. The models were built in R version 3.6.3.

## 3. Results

We obtained 135 responses from individuals who reported being diagnosed with MS, and Table 1 compares characteristics among PwMS and individuals diagnosed with arthritis (N = 586) and cancer (N = 622). PwMS were more likely to be women and have higher levels of education compared to the other groups. As might be expected, PwMS reported higher levels of spasticity relative to others but we ran no additional analyses given that only 21 of 135 PwMS identified spasticity as a reason that they used cannabis compared to only one or two people in the other groups. The majority of individuals from all three groups used cannabis to address pain and improve quality of sleep; persons with cancer were more likely to use it for digestive issues, as might be expected. PwMS reported lower levels of pain relative to persons with arthritis but higher levels than those with cancer, although they appeared far less likely to use prescription opioids than either group. PwMS appeared to differ from one or both groups across all five outcomes.

Our logistic regressions revealed that PwMS were less likely to report that cannabis increased productivity or exercise activities relative to persons with arthritis but generally experienced similar outcomes of using cannabis compared to those with arthritis or cancer (see Table 2). Persons who used cannabis for sleep or digestive problems regardless of diagnostic status experienced better outcomes across all five measures. The logistic regression models indicated that co-occurring use of prescription opioids contributed to poorer outcomes across all five measures.

## 4. Discussion

When PwMS were compared with groups of persons with arthritis or cancer who enrolled in the same state medical cannabis program, they were more likely to be female, as might be expected given that the incidence of MS is higher among females [1]. PwMS were quite similar otherwise and most of the older individuals who enrolled in the IMCP used cannabis primarily to manage symptoms of pain and improve sleep, whether they had MS, arthritis, or cancer.

PwMS initially appeared to fare more poorly in terms of wellbeing and all three movement-related activities of productivity, exercise, and social engagement. As such, we suspected that cannabis might have a more pronounced impact on improving these outcomes relative to persons with arthritis or cancer. However, our logistic regression modeling only suggests that persons with arthritis who used cannabis reported improved levels of productivity and exercise relative to PwMS, suggesting that the anti-inflammatory effect of cannabis may be more pronounced for arthritis or benefit those who may be less impaired at baseline.

Nevertheless, our results largely suggest that self-reported outcomes, especially those pertaining to psychological wellbeing and quality of life, are positively impacted by cannabis regardless of diagnostic condition. Cannabis also appears to assume a critical role in the lives of those who reported sleep problems, regardless of diagnostic condition. Taken together, these findings suggest that cannabis may positively alter psychoactive processes independently of any particular disease [14,15]. Moreover, rather than altering disease-specific mechanisms, our findings suggest that cannabis can positively improve digestive issues that occur among individuals regardless of diagnostic status, perhaps by working as an anti-inflammatory on digestive functions [15].

Our most striking finding concerned the pervasive negative impact of opioids. Persons who used opioids in the past year, regardless of diagnosis, simply did not report the same level of improvement across all five outcomes as those who only used cannabis. While the overall rate of opioid prescribing has trended downward in recent years, the decline remains smaller for older adults relative to younger age groups, and several public health concerns remain [23]. As pain most certainly continues to be a challenge, particularly for those over 60 with arthritis, cancer, and MS, our results suggest that cannabis may constitute a viable way to reduce or replace prescription opioids.

Researchers should dive deeper into the comparatively less frequent use of opioids among PwMS than persons with either arthritis or cancer. One possible explanation is how complementary and alternative approaches to pain management may be more accessible and normative for PwMS and they may use these along with cannabis as way to altogether avoid opioids to manage pain [5]. PwMS also may receive care from providers who may be less inclined to prescribe opioids [24] as the type of pain (neuropathic, spasticity, headaches) experienced by PwMS may be more amendable to cannabis or less responsive to opioids. The comparatively lower use of opioids among PwMS requires further consideration. Our findings suggest that persons with pain who used cannabis instead of opioids, regardless of whether they were diagnosed with arthritis, cancer, or MS, reported higher levels of psychological wellbeing, quality of life, productivity, exercise, and social engagement. Arguably, this is not a disease-specific effect as much as a reflection of a disease-specific approach to treatment.

### Limitations

Our results are limited to those who have a singular condition and comorbidities need to be addressed in future work. Although Illinois is one of the most populated states, our results may not be representative of all PwMS, and because enrollment into the ICMP was self-directed, our results may not even be applicable to other PwMS from Illinois. We recognize that given the self-selection, our study participants may have already held more favorable attitudes towards cannabis and therefore are more likely to report positive outcomes regardless of underlying diagnostic conditions. Moreover, because our research relied on a limited range of self-reported outcomes at a single point in time, we are cautious about reaching any conclusions about the effects that cannabis may have on these particular outcomes.

## 5. Conclusions

This study advanced the investigation of cannabis use among PwMS by comparing these individuals with others who enrolled in a state medical cannabis program and were qualified based on the diagnosis of arthritis or cancer. Our findings suggest that PwMS who use cannabis experience similar improvements in wellbeing and quality of life as those reported by persons with arthritis or cancer. This comparative evaluation, although limited in the number of groups and types of outcomes observed, suggests that cannabis mechanisms are not specific to MS, arthritis, or cancer as much as they impact processes that are common across the conditions. Perhaps more importantly, given the negative impact of opioids on self-reported outcomes, researchers should further explore why PwMS appear less likely to use prescription opioids than persons with arthritis or cancer, even though they experience comparable levels of pain.

## Figures and Tables

**Table 1:** Characteristics of respondents with arthritis, cancer, and MS.

	Arthritis	Cancer	MS	
N (%)	586 (43.6)	622 (46.3)	135 (10.1)	*p*-value
**Control variables—N (%)**				
Cannabis for sleep	395 (67.4)	415 (66.7)	78 (57.8)	0.094 ^2^
Cannabis for digestive issues	101 (17.2)	277 (44.5)	24 (17.8)	<0.001 ^1,3^
Cannabis for muscle spasticity	2 (0.3)	1 (0.2)	21 (15.6)	<0.001^2,3^
Female	321 (54.8)	279 (44.9)	88 (65.2)	<0.001 ^1,2,3^
Education (college and above)	238 (40.6)	11 (50.0)	75 (55.6)	<0.001 ^1,2^
Financial status *	398 (67.9)	469 (75.4)	95 (70.4)	0.015 ^1^
Past-year opioid use	293 (50.0)	258 (41.5)	45 (33.3)	<0.001 ^1,2^
Pain (mean (SD))	5.78 (2.09)	4.36 (2.49)	5.09 (2.30)	<0.001 ^1,2,3^
**Outcome variables—N (%)**				
Psychological wellbeing	395 (67.4)	402 (64.6)	74 (54.8)	0.022 ^2,3^
Quality of life	535 (91.3)	536 (86.2)	119 (88.1)	0.019 ^1^
Productivity	386 (65.9)	343 (55.1)	69 (51.1)	<0.001 ^1,2^
Exercise	271 (46.2)	227 (36.5)	42 (31.1)	<0.001 ^1,2^
Social satisfaction	222 (37.9)	208 (33.4)	36 (26.7)	0.032 ^2^

* Financial status is a binary variable which is coded as 1 if the respondent indicated a secure financial status and 0 otherwise. ^1^ Indicates that the difference between arthritis and cancer groups was significant at minimum 5% level of significance; ^2^ Indicates that the difference between arthritis and MS groups was significant at minimum 5% level of significance; ^3^ Indicates that the difference between cancer and MS groups was significant at minimum 5% level of significance.

**Table 2:** Logistic regression results showing relationship between cannabis use and self-reported outcomes of interest.

	Psychological Wellbeing	Quality of Life	Productivity	Exercise	Social Satisfaction
N	1343	1343	1343	1343	1343
Med Dx:					
MS	1.00 [Ref]	1.00 [Ref]	1.00 [Ref]	1.00 [Ref]	1.00 [Ref]
Cancer	1.12 [0.74, 1.71]	0.68 [0.37, 1.27]	1.16 [0.76, 1.77]	1.16 [0.74, 1.80]	1.11 [0.71, 1.75]
Arthritis	1.51 [1.00, 2.29]	1.51 [0.80, 2.84]	2.02 ** [1.33, 3.05]	1.86 ** [1.21, 2.86]	1.50 [0.96, 2.33]
Cannabis for sleep	1.42 ** [1.12, 1.81]	1.93 *** [1.36, 2.74]	1.52 *** [1.20, 1.93]	1.44 ** [1.13, 1.83]	1.35 * [1.06, 1.74]
Cannabis for digestive issues	1.82 *** [1.38, 2.40]	2.06 ** [1.34, 3.17]	1.74 *** [1.34, 2.27]	1.55 ** [1.20, 2.00]	1.59 *** [1.22, 2.06]
Cannabis for muscle spasticity	0.54 [0.22, 1.32]	3.03 [0.38, 24.53]	1.56 [0.62, 3.90]	0.99 [0.39, 2.53]	0.42 [0.13, 1.32]
Female	0.77 * [0.61, 0.97]	0.53 ** [0.37, 0.76]	1.16 [0.93, 1.46]	0.96 [0.77, 1.20]	0.97 [0.77, 1.22]
Education	0.90 [0.71, 1.13]	1.10 [0.77, 1.57]	0.90 [0.72, 1.13]	0.92 [0.73, 1.15]	0.85 [0.67, 1.08]
Financial status	0.86 [0.66, 1.13]	1.00 [0.67, 1.49]	1.03 [0.79, 1.33]	0.82 [0.64, 1.06]	0.85 [0.66, 1.11]
Past-year opioid use	0.77 * [0.60, 0.99]	0.51 *** [0.35, 0.74]	0.63 *** [0.49, 0.80]	0.75 * [0.59, 0.96]	0.70 ** [0.55, 0.90]
Pain	1.01 [0.96, 1.07]	1.04 [0.97, 1.13]	1.06 * [1.00, 1.12]	1.04 [0.99, 1.10]	1.02 [0.97, 1.08]
Constant	1.32 [0.74, 2.34]	6.62 *** [2.82, 15.54]	0.57 [0.33, 1.01]	0.38 ** [0.21, 0.68]	0.38 ** [0.21, 0.68]

Constant estimates baseline odds when all explanatory variables take on 0 value. * *p* < 0.05, ** *p* < 0.01, *** *p* < 0.001.

## Data Availability

The data presented in this study are available on request from the corresponding author.
